# Field Evaluation of a Portable Multi-Sensor Platform for Characterizing Inter-Individual Variability in Exhaled Breath Responses

**DOI:** 10.3390/s26206429

**Published:** 2026-10-12

**Authors:** Phillip Olla, Sara Shannan, Mina Maleki, Ava Travo, Mohammad Paknahad, Ava Bandi, Nina Favor

**Affiliations:** 1 Health Services Administration, College of Health Professions, University of Detroit Mercy, Detroit, MI 48221, USA; favornf@udmercy.edu; 2 Center for Augmenting Intelligence, University of Detroit Mercy, Detroit, MI 48221, USA; abandi@udmercy.edu; 3 Department of Electrical & Computer Engineering & Computer Science, University of Detroit Mercy, Detroit, MI 48221, USA; shannasa@udmercy.edu (S.S.); malekimi@udmercy.edu (M.M.); 4 London Health Sciences Centre, University of Western Ontario, London, ON N6A 3K7, Canada; avatravo8@gmail.com; 5 Cannabix Technologies Inc., 501-3292 Production Way, Burnaby, BC V5A 4R4, Canada; m.paknahad@cannabixtechnologies.com

**Keywords:** portable breath sensor, metal oxide semiconductor sensor, electronic nose, multi-sensor array, inter-individual variability

## Abstract

Portable breath-sensing platforms offer a practical approach for collecting physiological and environmental information outside controlled laboratory settings, but their interpretation is complicated by inter-individual variability and sensor-related effects. This pilot study evaluated the BreathLogix VOC Series prototype, a portable multi-sensor platform incorporating gas-sensitive, carbon-dioxide, temperature, and environmental channels, during routine collegiate athletic activities. A total of 421 breath recordings were obtained from 209 participant ID codes, including 175 pre-exercise and 246 post-exercise recordings. Continuous sensor signals were converted into aggregated sample-level features and analyzed using descriptive statistics, interquartile-range comparisons, Pearson correlation analysis, and K-means clustering. Group-level median responses remained relatively stable across exercise conditions, whereas response variability changed more noticeably. Humidity showed the largest post-exercise increase in dispersion, followed by the hydrogen- and methane-sensitive channels, while the acetone-sensitive response showed reduced variability. Correlation analysis identified strong relationships among selected channels, consistent with possible cross-sensitivity and environmental influence. Clustering revealed two overlapping sensor-derived breath-signature groups primarily separated by acetone-sensitive response; a sex difference in cluster composition was not retained when each participant was counted once. These findings demonstrate the feasibility of field-based multi-channel breath acquisition and highlight the value of variability-centered analysis. Further analytical validation, calibration assessment, and longitudinal reproducibility testing are required.

## 1. Introduction

Exhaled breath contains a complex mixture of volatile organic compounds (VOCs), respiratory gases, water vapor, and other components associated with physiological, metabolic, and environmental processes. Because breath collection is non-invasive and can be repeated with relatively little participant burden, breath analysis has attracted interest as a potential tool for health monitoring, physiological assessment, and longitudinal observation. However, the usefulness of breath-derived information depends not only on the biological relevance of the measured compounds but also on the performance, stability, and interpretability of the sensing platform used to acquire them [1,2,3].

Laboratory-based techniques such as gas chromatography–mass spectrometry (GC–MS) provide detailed identification and quantification of individual VOCs and remain important reference methods in breath research. Their routine use outside specialized laboratories is limited, however, by instrument cost, sample-handling requirements, analysis time, and the need for trained personnel [1,4]. Portable electronic-nose systems offer an alternative approach by using arrays of partially selective sensors to generate composite response patterns rather than identifying every compound individually. Metal oxide semiconductor (MOS) sensors are commonly used in these systems because of their sensitivity to gaseous compounds, relatively low cost, compatibility with miniaturization, and ability to support rapid multi-channel measurements [5,6].

Despite these practical advantages, MOS-based breath systems present important analytical challenges. Individual sensors may respond to multiple compounds, creating cross-sensitivity that complicates compound-specific interpretation. Sensor outputs may also be influenced by humidity, temperature, background air composition, baseline variation, and drift over time. Consequently, calibration procedures, environmental compensation, signal processing, and reference-method comparison are necessary when evaluating portable breath-sensing platforms. These limitations are especially relevant in field settings, where environmental conditions and participant breathing patterns are less controlled than in laboratory experiments [3,7,8].

A further challenge is the substantial inter-individual variability present in exhaled-breath profiles. Breath measurements may differ among individuals because of variation in metabolism, diet, circadian rhythms, sex, physical activity, respiratory mechanics, microbiome composition, and environmental exposure. This variability can obscure meaningful response patterns when analysis focuses only on group-level means or medians. Characterizing distribution spread and person-to-person heterogeneity is therefore important for distinguishing natural variation from changes associated with physiological stressors or disease processes [9,10,11].

Exercise provides a useful real-world context for examining this variability because it alters ventilation, substrate utilization, respiratory moisture, and other physiological processes that may affect multiple breath-sensor channels. Previous studies have reported exercise-associated changes in breath VOCs and respiratory gases, but many investigations have been performed using standardized laboratory protocols and analytical instrumentation [12,13,14]. Comparatively less is known about how portable multi-sensor systems perform across heterogeneous individuals during routine athletic activities, where exercise type, intensity, duration, and timing are not tightly controlled.

The present pilot study evaluated the BreathLogix VOC Series prototype, a portable multi-sensor breath-analysis platform, under field-based collegiate athletic conditions. Breath recordings were obtained from 209 participant ID codes; 182 athletes completed a demographic sign-up form and all 209 completed consent forms to participate in the study across multiple sports, producing a dataset of 421 pre- and post-exercise measurements. The study aimed to characterize group-level sensor-response distributions, quantify inter-individual variability, examine correlations among gas-sensitive, respiratory, and environmental channels, and identify exploratory sensor-derived breath-signature groups. The investigation was designed as a field evaluation of deployment feasibility and multi-channel response behavior rather than as a formal analytical or clinical validation of the device.

## 2. Materials and Methods

### 2.1. BreathLogix Multi-Sensor Platform

Breath samples were acquired using the BreathLogix VOC Series prototype (Cannabix Technologies Inc., Burnaby, BC, Canada), a portable electronic-nose platform designed for multi-channel analysis of exhaled breath. The prototype incorporated a heterogeneous sensor array consisting of four metal oxide semiconductor (MOS) gas sensors targeting methane, hydrogen, acetone, and broader air contaminant or volatile organic compound responses. The platform also included an STS21 digital I2C sensor for temperature and humidity, a Honeywell ABPDRRV001PDAA5 MEMS piezoresistive differential-pressure sensor, and an STC31-R3 thermal-conductivity carbon dioxide sensor. Together, these sensing elements enabled simultaneous acquisition of gas-sensitive, respiratory, and environmental characteristics of exhaled breath.

The gas-sensor array included the TGS2611-E00 methane sensor, TGS2616-C01 hydrogen sensor, TGS2600 air-contaminant and broader-VOC sensor, and TGS1820 acetone sensor. Temperature and humidity were measured using the STS21 digital I2C sensor based on CMOSens technology. Breath-pressure response was measured using the Honeywell ABPDRRV001PDAA5 analog MEMS piezoresistive differential-pressure sensor, while carbon dioxide was measured using the STC31-R3 thermal-conductivity CMOSens sensor. The sensor components and their intended response channels are summarized in Table 1.

Breath sampling was performed actively using an integrated diaphragm pump that drew exhaled breath through the sensor chamber. The pump-controlled flow rate was approximately 8 L/min, and the target sampling volume was approximately 1 L of exhaled breath per measurement. Participants provided a continuous exhalation through a single-use plastic straw connected to the sampling inlet until a sufficient sample volume was collected.

Sensor signals were acquired at 10 Hz per channel during breath collection. The prototype used Raspberry Pi-based embedded processing and was powered through an external 12 V DC supply. The system measured approximately 17 × 16 × 7 cm and weighed approximately 2 kg. Available connectivity included Wi-Fi, Ethernet when enabled, and cloud synchronization. The principal system-level specifications are summarized in Table 2.

### 2.2. Study Population and Design

This pilot observational study was conducted at the University of Detroit Mercy during the 2025 athletic year (spring and fall sessions). Collegiate student-athletes participating in NCAA-sanctioned sports were recruited from multiple athletic programs and voluntarily enrolled in the study.

A total of 182 athletes between 17 and 26 years of age participated, including 98 males and 84 females. Eligible participants were actively engaged in athletic training and competition and were free from acute illness at the time of data collection. The study protocol was approved by the Institutional Review Board of the University of Detroit Mercy (IRB Protocol No. 23-24-80; Protocol ID 232480; approved 18 March 2024), and all participants provided informed consent before participation.

Breath samples were collected under field-based athletic training conditions in spring and fall 2025. Participants provided breath samples before exercise, after exercise, or under both conditions. In total, 421 breath recordings were obtained from 209 participant ID codes, including 175 pre-exercise recordings and 246 post-exercise recordings. Of these, 161 ID codes (338 recordings) matched one of the 182 completed sign-up forms; one matched ID code was shared by two sign-up forms and was excluded from demographic analyses, leaving 160 participants (329 recordings) with linked sex and sport information. The remaining 48 ID codes (83 recordings) could not be matched to a sign-up form. All 421 recordings were used in sensor-response, variability, correlation, and clustering analyses.

Repeated measurements were common: 141 participants contributed one recording and 68 contributed more than one (maximum 19). Forty-four participants contributed both pre- and post-exercise recordings, 77 contributed pre-exercise recordings only, and 88 contributed post-exercise recordings only. Because recordings from the same participant are not independent, the variability reported here includes both between-participant and within-participant components. Participants also completed brief questionnaires assessing sleep quality, perceived readiness to train, meal timing, hydration timing, and workout status. These variables were retained as participant-level metadata and examined in relation to the multi-sensor breath-response patterns.

Given the exploratory nature of the study, the use of a prototype breath analysis platform, and the absence of a standardized exercise intervention, the investigation was designed as a pilot, hypothesis-generating field evaluation. The study aimed to characterize inter-individual variability in multi-sensor breath responses and explore whether discriminable breath-signature groups could be identified within the athlete’s cohort.

### 2.3. Breath Sample Collection

Before each breath collection event, participants entered a unique study identification code into the BreathLogix interface and completed a brief touchscreen questionnaire. The questionnaire recorded workout status and self-reported information related to sleep quality, perceived readiness to train, time since the last meal, and time since the last fluid intake.

Participants then provided a sustained, continuous exhalation through a single-use plastic straw connected to the device sampling inlet. During exhalation, the integrated diaphragm pump actively drew the breath sample through the sensor chamber at an approximate pump-controlled flow rate of 8 L/min. Participants continued exhaling until the system collected approximately 1 L of breath and indicated that sufficient sample volume had been obtained. Each exported recording spans approximately 110 s (range 108 to 123 s) and represents the full time series, comprising a pre-exhalation baseline, the breath-sampling period, and a post-exhalation sensor-response (recovery) period. The breath-sampling period itself lasted approximately 5–6 s, during which approximately 1 L of exhaled breath was delivered to the sensor chamber.

The sensor channels were recorded simultaneously throughout each breath collection event, producing continuous time-series measurements for subsequent processing and analysis. A new disposable plastic straw was used for each participant to reduce the risk of cross-contamination between collection events.

Breath samples were collected in field-based athletic environments rather than under controlled laboratory conditions. Samples were obtained before exercise, after exercise, or under both conditions, depending on participant availability and training schedules. No standardized post-exercise sampling window was implemented because practice and game timings varied across teams and sessions. The interval between the end of exercise and post-exercise sampling was therefore not recorded for individual participants. This field-based protocol was intended to evaluate the practical deployment of the multi-sensor platform during routine collegiate athletic activities.

### 2.4. Sensor Data Acquisition and On-Board Processing

During each breath collection event, all sensor channels were sampled simultaneously at 10 Hz. The resulting multi-channel time-series data were processed by Raspberry Pi-based embedded firmware integrated into the BreathLogix prototype.

Sensors were warmed up for 30 min before each session. The on-board processing workflow then included sensor warm-up verification, baseline correction, moving-average filtering, and temperature compensation [7], and breath-phase detection using the carbon dioxide channel. These processing steps were intended to improve signal stability, reduce short-term noise, and identify the portion of each recording corresponding to the active breath sample.

The device firmware also supported extraction of signal-response features, including peak response, response slope, and area under the curve, followed by multi-sensor fusion before downstream analysis. However, the clustering and statistical analyses reported in the present study were based on aggregated sample-level features rather than these firmware-derived signal features, as described in Section 2.5. Because the response times of MOS sensors (several seconds) are slower than a single exhalation, summary statistics were calculated over the full recording window rather than dynamic response characteristics.

### 2.5. Feature Extraction and Data Preparation

Each breath recording consisted of continuous time-series measurements from the available sensor channels. To transform these recordings into a structured dataset suitable for statistical analysis, five summary features were calculated for each measured variable: mean, median, minimum, maximum, and standard deviation. These features represented the central tendency, range, and within-recording variability of the sensor responses while reducing the dimensionality of the raw time-series data. The interquartile-range analyses used the per-recording mean of each channel, and the clustering used the per-recording median and maximum values specified in Section 2.6.4.

Following aggregation, each row of the analytical dataset represented one breath recording. Participant metadata, including study identification code, exercise condition, sex, sport, and questionnaire responses, were retained and linked to the corresponding sample-level sensor features.

Because the sensor channels were recorded on substantially different numerical scales, continuous features were normalized before correlation and multivariate analyses. Min–max normalization was applied according to

*x*_norm_ = (*x* − *x*_min_)/(*x*_max_ − *x*_min_) (1)

where x represents the original feature value and xmin and xmax represent the minimum and maximum observed values, respectively, for the corresponding feature. This transformation scaled each continuous feature to a common range while preserving the relative differences among observations.

Before statistical analysis, the aggregated dataset was reviewed for completeness and consistency. Recordings with incomplete sensor data or missing information required for the relevant analysis were excluded. The contaminant channel and other contextual sensor variables were retained to assist with the interpretation of unusual measurements and potential sensor-related variation.

### 2.6. Statistical Analysis

Statistical analyses were performed to characterize pre- and post-exercise sensor-response distributions, quantify inter-individual variability, identify unusual observations, evaluate relationships among sensor channels, and explore sensor-derived breath-signature groups. Analyses included descriptive statistics, interquartile-range comparisons, outlier detection, Pearson correlation analysis, unsupervised K-means clustering, and comparisons of cluster composition by sex and sport.

#### 2.6.1. Descriptive and Variability Analysis

Sensor-response distributions were examined separately for pre-exercise and post-exercise recordings. Median values and interquartile ranges (IQRs) were calculated for each primary sensor feature to characterize central tendency and distribution spread. Exercise-associated changes in variability were quantified using the ratio of the post-exercise IQR to the pre-exercise IQR:

IQR Ratio = IQR_post_/IQR_pre_ (2)

An IQR ratio greater than 1 indicated greater variability among post-exercise recordings, whereas a ratio less than 1 indicated reduced post-exercise variability. Because participants did not necessarily contribute recordings under both conditions, these comparisons were performed at the recording level and were not treated as fully paired pre–post comparisons. In this study, “inter-individual variability” therefore refers to variability among recordings, which includes both between-participant and within-participant variation (Section 2.2).

#### 2.6.2. Outlier Analysis

Outliers were identified separately within the pre-exercise and post-exercise groups using the standard IQR rule originally described by Tukey [15]. For each sensor feature, the lower and upper thresholds were defined as

Lower Bound = Q1 − 1.5 × IQR(3)

and

Upper Bound = Q3 + 1.5 × IQR(4)

where

IQR = Q3 − Q1(5)

Recordings falling outside these thresholds were classified as feature-specific outliers. Extreme observations were retained unless they were attributable to incomplete recordings or identifiable technical errors because they could represent either meaningful inter-individual responses or sensor-related variation.

#### 2.6.3. Correlation Analysis

Pearson correlation coefficients were calculated using normalized sample-level sensor features to evaluate linear relationships among the gas-sensitive, respiratory, and environmental channels. Correlation matrices were generated as an exploratory tool to identify shared response patterns and possible interactions among sensor channels. These associations were interpreted as relationships among measured sensor features and not as confirmation of direct physiological or compound-specific mechanisms.

#### 2.6.4. Sensor-Derived Breath-Signature Clustering Analysis

Unsupervised K-means clustering was used to identify natural groupings within the aggregated sensor-feature space. The clustering analysis used maximum pressure, median pressure, median acetone, and median carbon dioxide as the primary input features. Features were normalized before clustering to reduce the influence of differences in numerical scale.

The number of clusters was evaluated using silhouette analysis [16], within-cluster sum of squares, and entropy-based assessments of within-cluster disorder and between-cluster separation. Clustering was applied to all 421 recordings, using k-means with 10 random starts (random seed 42). Average silhouette widths were low (0.25 for k = 2, 0.27 for k = 3, and 0.28 for k = 4); a two-cluster solution (k = 2) was retained as the most interpretable exploratory grouping, and cluster labels were stable across 10 random seeds (minimum agreement 97.6%). Cluster assignments were subsequently examined in relation to exercise condition, sex, sport participation, meal timing, and hydration timing. The resulting groups were interpreted as exploratory sensor-derived breath-signature clusters rather than definitive physiological phenotypes.

#### 2.6.5. Sex- and Sport-Based Comparisons

Cluster composition by sex was compared with a chi-square test on a 2 × 2 table (Fisher’s exact test as a check), using the recordings that could be linked to sign-up information (*n* = 329 recordings from 160 participants). Because recordings from the same participant are not independent, the sex comparison was repeated using one recording per participant (the first recording; *n* = 160). Sport composition is reported as the percentage of each cluster, with one overall chi-square test of association; no per-sport tests were performed because several sport categories had small counts. Statistical significance was defined as *p* < 0.05. Because the analyses were exploratory, the findings were interpreted as hypothesis-generating rather than confirmatory.

## 3. Results

### 3.1. Dataset and Field Deployment

Breath recordings were obtained from 209 participant ID codes. The sign-up sheet contained 182 completed forms from collegiate athletes, including 98 men (53.8%) and 84 women (46.2%), aged 17 to 26 years, representing multiple NCAA athletic programs; 160 participants (329 recordings) could be linked unambiguously to a sign-up form (Section 2.2). The final analytical dataset comprised 421 breath recordings, including 175 pre-exercise recordings and 246 post-exercise recordings. Participants contributed pre-exercise recordings, post-exercise recordings, or recordings under both conditions, depending on availability and training schedules.

The sign-up forms listed athletes from Track & Field/Cross Country (*n* = 50), Soccer (*n* = 40), Lacrosse (*n* = 33), Basketball (*n* = 31), Softball (*n* = 17), Fencing (*n* = 7), and other sports (*n* = 4). Participant and dataset characteristics are summarized in Table 3.

### 3.2. Pre- and Post-Exercise Sensor-Response Distributions

**Figure 1:**
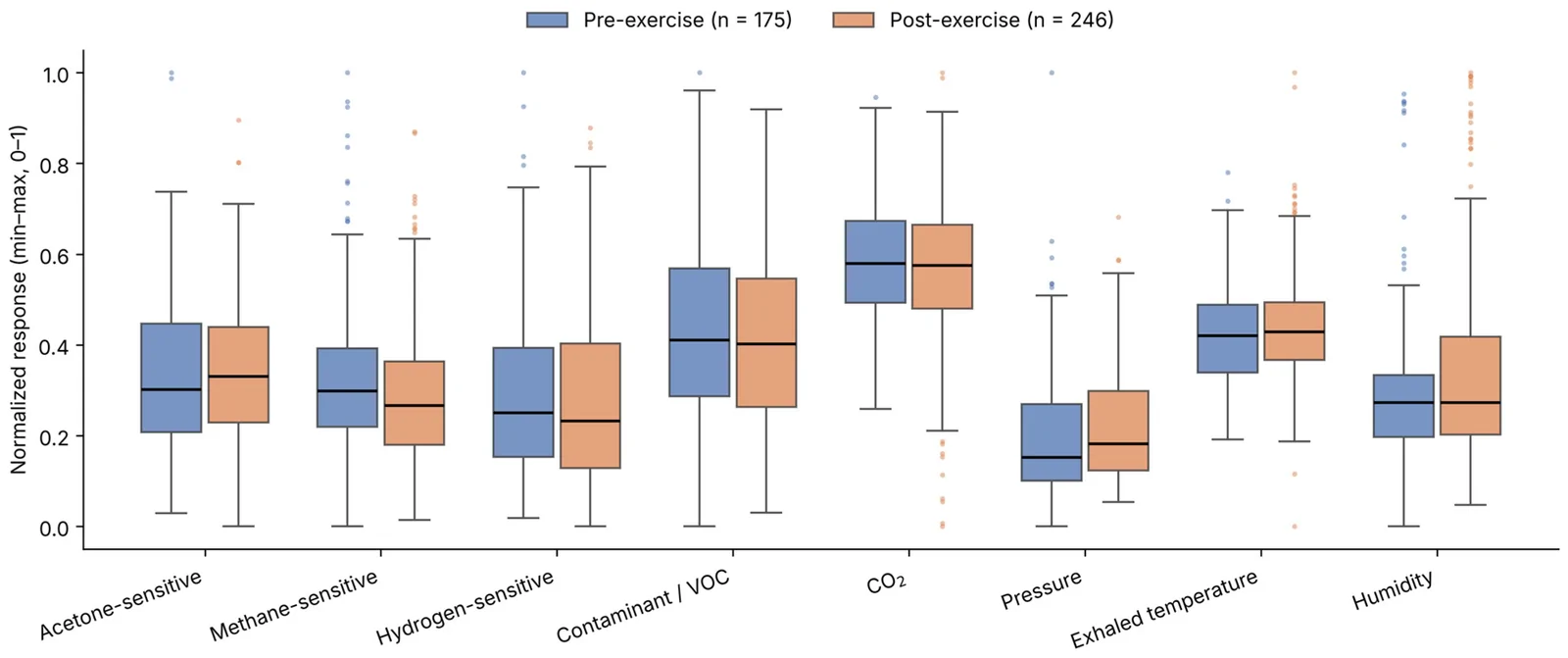
Normalized distributions of gas-sensitive, respiratory, and environmental sensor features in pre-exercise and post-exercise recordings. Boxes represent the interquartile range, center lines represent the median, and individual markers indicate observations outside the whisker range. Values are per-recording means scaled to 0–1 (Equation (1)); *n* = 175 pre-exercise and 246 post-exercise recordings.

Sensor-response distributions were compared between the 175 pre-exercise recordings and 246 post-exercise recordings. Figure 1 summarizes the normalized distributions of the primary gas-sensitive, respiratory, and environmental features across both conditions.

Across most sensor channels, median responses remained relatively stable between pre-exercise and post-exercise recordings. The acetone, methane, hydrogen, carbon dioxide, contaminant, pressure, and exhaled temperature features exhibited only modest shifts in central tendency, indicating that exercise condition was not associated with a large, uniform change in the group-level sensor response (for example, the median of the per-recording mean CO_2_ response changed by about −0.5%, from 38,596 to 38,408 device units).

Humidity showed the most noticeable difference between conditions. Post-exercise recordings exhibited a broader distribution of humidity response than pre-exercise recordings (IQR ratio 1.58), whereas the median was essentially unchanged (27.59 vs. 27.58% RH); the mean was about 5% higher post-exercise (30.0 vs. 28.5% RH), reflecting a longer upper tail. This pattern is consistent with increased respiratory moisture following physical activity, although humidity may also influence the response of MOS gas sensors and should therefore be interpreted as both a physiological variable and a potential environmental-interference factor.

Overall, the pre- and post-exercise comparison suggested that the platform captured more pronounced changes in response dispersion than in group-level median values. This observation motivated the variability-centered analysis presented in the following subsection.

### 3.3. Inter-Individual Variability in Sensor Responses Quantification 

**Figure 2:**
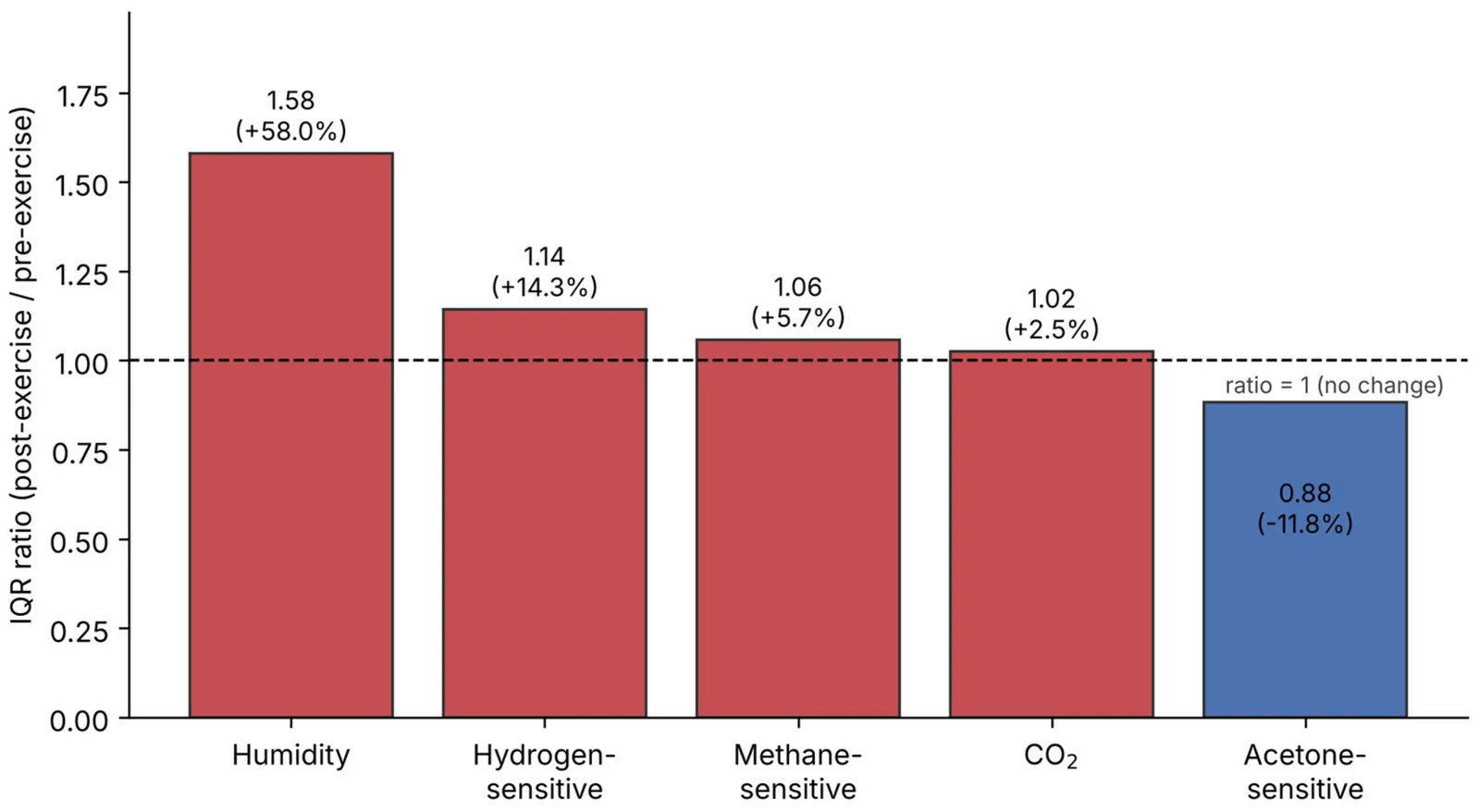
Post-exercise to pre-exercise interquartile-range ratios.

Inter-individual variability was quantified using the interquartile range for each primary sensor feature in the pre-exercise and post-exercise groups, as seen in Figure 2. Relative changes in variability were expressed as the ratio of the post-exercise IQR to the pre-exercise IQR, where values greater than 1 indicated increased post-exercise dispersion and values less than 1 indicated reduced dispersion.

Four of the five primary features showed greater variability in the post-exercise recordings. Humidity exhibited the largest relative increase, with an IQR ratio of 1.58, corresponding to a 58.01% increase in distribution spread. Hydrogen showed the second-largest increase, with an IQR ratio of 1.14 (+14.31%), followed by methane with an IQR ratio of 1.06 (+5.75%) and carbon dioxide with an IQR ratio of 1.02 (+2.47%). In contrast, the acetone-sensitive response showed reduced post-exercise variability, with an IQR ratio of 0.88 (−11.77%).

These findings indicate that the most notable exercise-associated changes were reflected in the spread of sensor responses rather than in uniform shifts in their median values. The magnitude of the variability change differed across channels, suggesting that the platform captured heterogeneous response patterns among athletes under field-based exercise conditions.

Humidity and hydrogen displayed the largest increases in dispersion, whereas the acetone-sensitive channel became slightly more uniform in the post-exercise group. Because the analysis compared recordings at the group level and was not a fully paired pre–post design, these results are interpreted as differences associated with exercise condition rather than as within-person causal changes.

### 3.4. Correlations Among Sensor Channels

**Figure 3:**
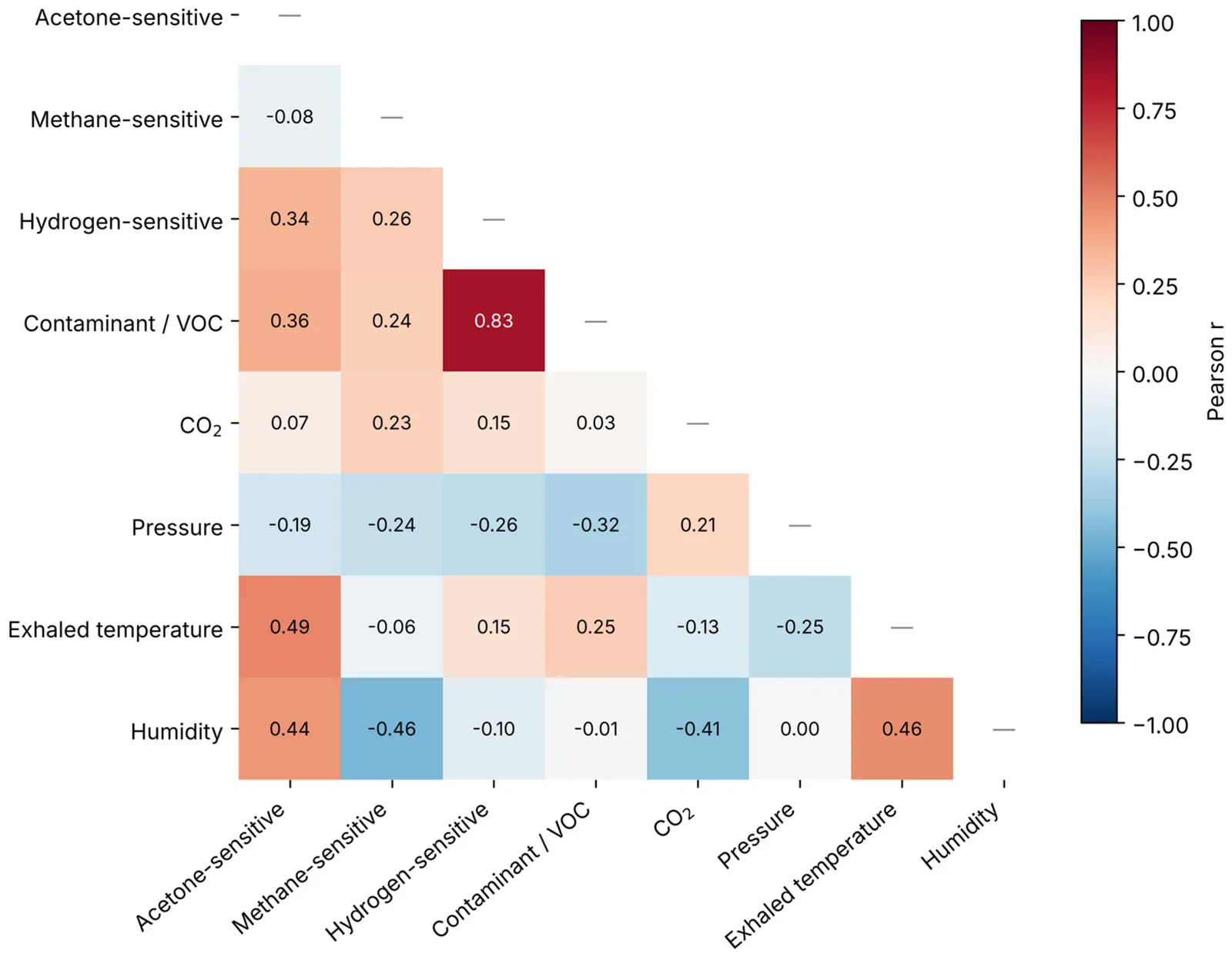
Pearson correlation matrix.

Pearson correlation analysis was used to examine relationships among the normalized gas-sensitive, respiratory, and environmental sensor features. The resulting correlation matrix is shown in Figure 3.

The strongest positive association was observed between the hydrogen-sensitive and contaminant channels (r = 0.83). Because both channels were obtained from sensors that may respond to overlapping compounds or shared environmental conditions, this relationship may reflect cross-sensitivity or common signal interference rather than a direct biological association.

The acetone-sensitive response showed moderate positive correlations with exhaled temperature (r = 0.49) and humidity (r = 0.44). Conversely, humidity was negatively correlated with both the carbon-dioxide response (r = −0.41) and methane-sensitive response (r = −0.46). These relationships indicate that moisture and temperature may have influenced multiple channels and should be considered when interpreting breath-sensor measurements collected under post-exercise field conditions.

Acetone-sensitive response, carbon dioxide, and pressure showed weak pairwise relationships, with absolute correlation coefficients below 0.30. More broadly, the respiratory and environmental channels tended to show stronger interrelationships than the gas-sensitive channels. The generally weaker correlations among several gas-sensitive features suggest that they contributed partially independent information to the multi-sensor breath profiles.

Because these analyses were exploratory, the observed correlations were interpreted as relationships among sensor outputs rather than as evidence of chemically specific or causal physiological mechanisms.

### 3.5. Sensor-Derived Breath-Signature Clustering

**Figure 4:**
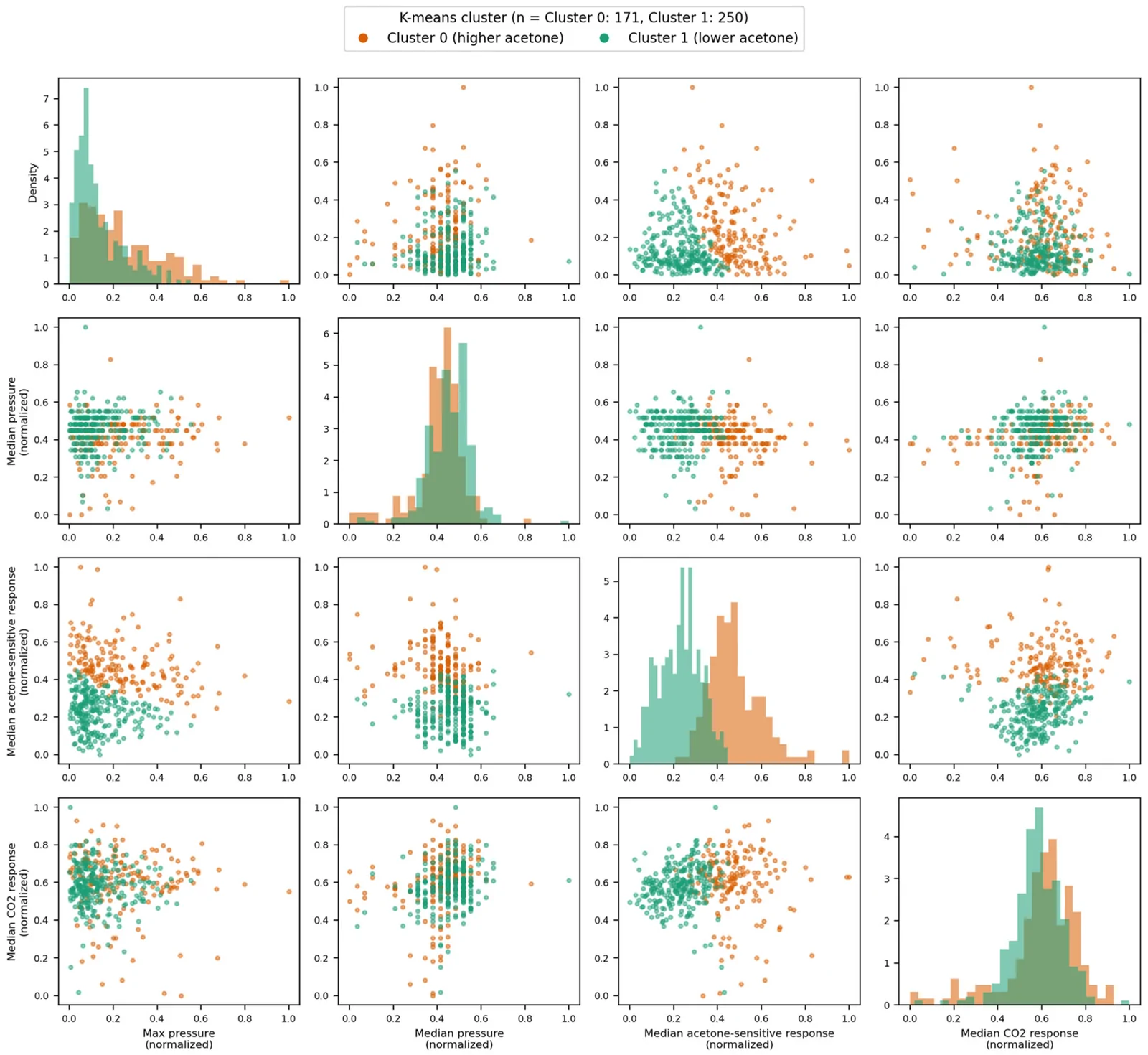
Pairwise distributions of maximum pressure, median pressure, median acetone-sensitive response, and median carbon-dioxide response stratified by K-means cluster assignment. Diagonal panels show the distribution of each individual feature, while off-diagonal panels show pairwise relationships between features. Cluster 0 (*n* = 171) represents the higher acetone-sensitive-response group, and Cluster 1 (*n* = 250) represents the lower acetone-sensitive-response group. The strongest separation occurred along the median acetone-sensitive-response dimension, while substantial overlap remained across the other feature combinations.

Unsupervised K-means clustering was applied to the normalized sample-level features to identify natural groupings within the multi-sensor breath dataset. The clustering analysis included maximum pressure, median pressure, median acetone-sensitive response, and median carbon-dioxide response. Cluster separation was modest (average silhouette width 0.25 for k = 2; slightly higher values for k = 3 and k = 4, 0.27 and 0.28), and k = 2 was retained as an interpretable exploratory solution. Cluster 0 (higher acetone-sensitive response) contained 171 recordings and Cluster 1 (lower acetone-sensitive response) contained 250 recordings. Both clusters included pre-exercise and post-exercise recordings (Cluster 0: 70 pre, 101 post; Cluster 1: 105 pre, 145 post). Figure 4 presents the pairwise distributions of these four clustering features. The diagonal panels show the distribution of each individual feature within the two clusters, whereas the off-diagonal panels show the pairwise relationships between features. Across the diagonal panels, the clearest difference between the clusters occurred for median acetone-sensitive response. Cluster 0 displayed a broader distribution extending toward higher values, whereas Cluster 1 showed a narrower distribution concentrated at lower values.

The off-diagonal panels similarly showed that separation between the clusters was most visible in feature combinations that included median acetone-sensitive response. For this reason, Cluster 0 is referred to as the higher acetone-sensitive-response group and Cluster 1 as the lower acetone-sensitive-response group.

Median pressure also contributed to cluster differentiation, with the lower acetone-sensitive-response group displaying a more tightly concentrated pressure distribution. In contrast, the pairwise plots involving median carbon dioxide and maximum pressure showed substantial overlap between clusters, indicating that these features contributed less strongly to group separation.

Overall, the considerable overlap observed across several off-diagonal panels indicates that the two groups were not sharply separated across the complete feature space. The clusters are therefore interpreted as exploratory sensor-derived breath-signature groups within a continuous multi-sensor response distribution rather than as biologically definitive phenotypes.

### 3.6. Associations with Sex and Sport

Among recordings linked to sign-up information (*n* = 329), men accounted for 71.1% of Cluster 0 (86 of 121) and 48.1% of Cluster 1 (100 of 208), whereas women accounted for 28.9% (35 of 121) and 51.9% (108 of 208), respectively (chi-square *p* < 0.001; Fisher’s exact *p* < 0.001; Table 4). This association was not retained when only one recording per participant was analyzed (*n* = 160 participants; Fisher’s exact *p* = 0.10), suggesting that it may be partly driven by repeated recordings from the same individuals.

Sport composition is summarized in Table 5. Track & Field/Cross Country athletes represented 24.0% of Cluster 0 and 35.1% of Cluster 1, and soccer athletes 36.4% and 28.4%; the other sports differed by less than three percentage points. There was no overall association between sport and cluster membership (chi-square *p* = 0.39; *p* = 0.93 with one recording per participant), although several sport categories had small counts.

These findings should not be interpreted as evidence that sex or sport determined sensor response because cluster membership may also have been influenced by training status, recent exercise, nutritional state, respiratory mechanics, repeated recordings, and sensor-related variability.

## 4. Discussion

### 4.1. Field Performance of the Multi-Sensor Platform

The present study demonstrated the practical feasibility of deploying the BreathLogix multi-sensor platform in a field-based collegiate athletic setting. The prototype could be used across a relatively large and heterogeneous cohort during routine training activities outside a controlled laboratory environment.

Several design features supported this field deployment. The active sampling system used an integrated diaphragm pump to draw approximately 1 L of exhaled breath through the sensing chamber at a pump-controlled flow rate of approximately 8 L/min. Compared with a purely passive sampling configuration, this approach provided a device-controlled mechanism for transporting each breath sample across the sensor array. The single-use plastic straw also offered a simple breath interface that could be replaced between participants, supporting repeated sample collection in a shared athletic setting.

The simultaneous acquisition of multiple channels was another practical advantage of the platform. Gas-sensitive, carbon-dioxide, temperature, humidity, and pressure signals were recorded together at 10 Hz, allowing each breath event to be represented as a multi-channel time-series measurement rather than as an isolated single-sensor reading. This configuration enabled the subsequent examination of response distributions, inter-individual variability, channel correlations, and sensor-derived breath-signature groups within the same dataset.

These findings should be interpreted as evidence of deployment feasibility rather than formal analytical validation. The study did not include concurrent reference measurements, controlled calibration-gas testing, or formal assessments of sensitivity, selectivity, limits of detection, or long-term reproducibility [1,17]. Therefore, the successful collection of field-based recordings demonstrates that the platform was operational and practically usable in this setting, but it does not by itself establish the analytical accuracy of the individual sensor channels.

### 4.2. Inter-Individual Variability in Sensor Responses

A central finding of this study was that group-level median sensor responses remained relatively stable between pre-exercise and post-exercise recordings, while the spread of several response channels changed more noticeably. This pattern indicates that exercise-related differences were not expressed as a single uniform shift across the athlete cohort. Instead, the platform captured heterogeneous responses that varied across individuals and sensor channels.

The largest increase in post-exercise variability was observed for humidity, followed by the hydrogen-sensitive and methane-sensitive channels. Carbon dioxide showed only a small increase in dispersion, whereas the acetone-sensitive response showed a modest reduction in variability. These channel-specific differences suggest that the physiological and environmental effects associated with exercise were not reflected equally across the sensor array.

The variability-centered analysis is important because group averages can obscure substantial person-to-person differences [17,18]. Two groups may show similar median responses while containing individuals with markedly different post-exercise patterns. In this dataset, the broader post-exercise distributions for selected channels suggest that athletes did not respond uniformly under field conditions, potentially because of differences in exercise type, intensity, duration, recent nutrition, hydration, respiratory mechanics, and training status.

These findings should nevertheless be interpreted cautiously. The pre-exercise and post-exercise groups were not fully paired, and some participants may have contributed more than one recording. Therefore, the observed differences in variability represent recording-level group patterns rather than confirmed within-person changes. A longitudinal design with standardized exercise conditions and repeated paired measurements would be required to determine whether the observed variability reflects stable individual response characteristics or short-term measurement and contextual effects.

### 4.3. Value and Limitations of Multi-Sensor Integration

The use of a heterogeneous sensor array allowed the BreathLogix platform to capture complementary gas-sensitive, respiratory, and environmental responses within the same breath event. Acetone-, methane-, hydrogen-, and contaminant-related channels were recorded alongside carbon dioxide, temperature, humidity, and pressure, providing a broader representation of each sample than would be available from a single-sensor system.

This multi-channel configuration was particularly useful for examining relationships among sensor outputs. The observed correlations showed that some channels contributed partially independent information, whereas others were more strongly associated. For example, the strong relationship between the hydrogen-sensitive and contaminant channels may reflect overlapping sensor sensitivity, shared environmental influence, or common signal interference. Similarly, the associations between humidity, temperature, and several gas-sensitive channels indicate that environmental and respiratory conditions may affect the measured response profile.

These findings highlight both the value and the challenge of multi-sensor integration. Combining several sensing technologies may improve the ability to characterize complex breath-response patterns, but it also complicates interpretation because MOS sensors are not perfectly selective for a single analyte. Cross-sensitivity, baseline variation, temperature dependence, and humidity interference can alter sensor output and may contribute to apparent differences among samples [6,7].

The on-board use of warm-up verification, baseline correction, moving-average filtering, and temperature compensation was intended to improve signal stability under field conditions. However, these processing steps do not eliminate the possibility of sensor drift, channel interaction, or residual environmental effects. The present correlation results therefore support interpreting the recorded variables as sensor-response features rather than as fully validated compound-specific concentrations.

Future studies should evaluate the individual channels against reference analytical methods and controlled gas standards [17]. Repeated calibration experiments, humidity- and temperature-controlled testing, and longitudinal drift assessment would help determine the extent to which the observed multi-sensor patterns represent physiological breath variation versus device-related response variation.

### 4.4. Sensor-Derived Breath-Signature Groups

The clustering analysis identified two broad sensor-derived breath-signature groups within the normalized feature space. The clearest separation occurred along the median acetone-sensitive-response dimension, while median pressure contributed more modestly and the carbon-dioxide and maximum-pressure features showed substantial overlap between groups.

These results suggest that the multi-sensor platform captured recurring response patterns that were not explained solely by acute exercise status. Both clusters included pre-exercise and post-exercise recordings, indicating that cluster membership was not simply a direct reflection of whether a sample had been collected before or after exercise.

The groups differed in sex composition at the recording level, but this association was not retained when each participant was counted once, and no overall association with sport was observed; participant characteristics therefore cannot be considered established determinants of cluster membership. However, these relationships should not be interpreted as proof that the clusters represent distinct metabolic phenotypes [19]. Cluster membership may also have been influenced by respiratory mechanics, recent nutrition, training state, environmental conditions, repeated measurements, and sensor-related variation.

The substantial overlap visible across several pairwise feature combinations supports interpreting the two groups as exploratory stratifications within a continuous response distribution rather than as sharply separated biological classes. Future studies incorporating reference biomarkers, standardized exercise conditions, and repeated within-participant measurements will be needed to determine whether similar response groups are reproducible and physiologically meaningful.

### 4.5. Limitations and Future Validation

This pilot study has several limitations. First, the BreathLogix prototype was evaluated in a field setting without concurrent gas-chromatography–mass-spectrometry measurements, controlled gas standards, blood-ketone measurements, or metabolic-cart testing [1,17]. Accordingly, the recorded channels should be interpreted as sensor-response features rather than analytically validated compound-specific concentrations.

Second, the MOS channels may have been affected by cross-sensitivity, humidity, temperature, baseline variation, and sensor drift [7]. Although the device incorporated warm-up verification, baseline correction, filtering, and temperature compensation, the study did not include formal assessments of limits of detection, analytical sensitivity, selectivity, calibration stability, or long-term drift across the data collection period.

Third, exercise conditions were not standardized with respect to type, duration, intensity, or timing of post-exercise sampling. The numbers of pre-exercise and post-exercise recordings were unequal, and the analysis was not based on a fully paired design. Many athletes contributed repeated recordings (68 of 209 participant ID codes contributed more than one, and only 44 contributed both pre- and post-exercise recordings), which affects the independence of observations and means that the reported variability includes within-participant variation. The interval between the end of exercise and post-exercise sampling was not recorded for individual participants, and demographic information could be linked to 182 of the 209 participant ID codes.

Fourth, the study was conducted at a single institution and included athletes from unevenly represented sport categories. Self-reported meal timing, hydration timing, sleep quality, and readiness measures were not supported by controlled dietary or physiological measurements. The clustering analysis was exploratory, and the resulting groups were not validated in an independent cohort.

Future work should include controlled calibration experiments, reference-method comparison, repeated-session reproducibility testing, and longitudinal evaluation of sensor drift. Standardized exercise and breath-collection protocols, paired within-participant sampling, and independent validation cohorts would strengthen the interpretation of the observed response patterns.

## 5. Conclusions

This pilot study demonstrated the practical feasibility of using the BreathLogix portable multi-sensor platform to acquire multi-channel exhaled-breath recordings from collegiate athletes under field-based training conditions. The system could be deployed outside a controlled laboratory environment while simultaneously capturing gas-sensitive, respiratory, temperature, and humidity-related sensor responses.

The principal findings were characterized more strongly by differences in response variability than by uniform changes in group-level median values. Post-exercise recordings showed greater dispersion in several channels, particularly humidity and the hydrogen-sensitive response, while the acetone-sensitive channel showed slightly reduced variability. Correlation and clustering analyses further indicated that the platform captured heterogeneous multi-sensor response patterns across the athlete cohort.

These findings support the value of a variability-centered approach for interpreting field-based breath-sensor data, where person-to-person differences may be obscured by group averages. However, the results should not be interpreted as clinical validation of the device or as evidence of definitive physiological phenotypes. Further work is needed to assess analytical accuracy, calibration stability, cross-sensitivity, environmental interference, sensor drift, and repeated-session reproducibility using controlled protocols and appropriate reference methods.

Overall, the study provides a foundation for continued evaluation of portable multi-sensor breath platforms in real-world monitoring applications and highlights the importance of combining field feasibility testing with rigorous analytical validation.

## Figures and Tables

**Table 1:** Sensor components incorporated into the BreathLogix prototype.

Sensor Model	Intended Response Channel	Sensor Technology
TGS2611-E00	Methane-sensitive response	Metal oxide semiconductor
TGS2616-C01	Hydrogen-sensitive response	Metal oxide semiconductor
TGS2600	Air contaminants and broader VOC response	Metal oxide semiconductor
TGS1820	Acetone-sensitive response	Metal oxide semiconductor
STS21	Temperature and humidity	Digital I2C CMOSens sensor
Honeywell ABPDRRV001PDAA5	Differential pressure	MEMS piezoresistive analog pressure sensor
STC31-R3	Carbon dioxide	Thermal-conductivity CMOSens sensor

**Table 2:** System-level specifications of the BreathLogix prototype.

Parameter	Prototype Specification
Sampling method	Active sampling using an integrated diaphragm pump
Approximate flow rate	8 L/min, pump-controlled
Approximate sampling volume	1 L of exhaled breath per measurement
Breath interface	Single-use plastic straw connected to the sampling inlet
Data-acquisition rate	10 Hz per sensor channel
On-board processor	Raspberry Pi-based embedded processing system
Approximate dimensions	17 × 16 × 7 cm
Approximate weight	2 kg
Power supply	External 12 V DC supply
Connectivity	Wi-Fi, Ethernet when enabled, and cloud synchronization

**Table 3:** Characteristics of the 182 sign-up forms and the breath dataset.

Characteristic	Value
Sign-up forms	182 (209 participant ID codes in breath data; 160 linked)
Male	98 (53.8%)
Female	84 (46.2%)
Age range, years	17–26
Total breath recordings	421
Pre-exercise recordings	175
Post-exercise recordings	246
Basketball	31
Fencing	7
Lacrosse	33
Other sports	4
Soccer	40
Softball	17
Track & Field/Cross Country	50

**Table 4:** Sex composition of the two sensor-derived breath-signature clusters (linked recordings, *n* = 329).

Sex	Cluster 0, *n* (%)	Cluster 1, *n* (%)
Male	86 (71.1)	100 (48.1)
Female	35 (28.9)	108 (51.9)
Total	121	208

* Chi-square *p* < 0.001; Fisher’s exact *p* < 0.001. With one recording per participant (*n* = 160), Fisher’s exact *p* = 0.10.

**Table 5:** Sport composition of the two sensor-derived breath-signature clusters (linked recordings, *n* = 329).

Sport	Cluster 0, *n* (%)	Cluster 1, *n* (%)
Track & Field/Cross Country	29 (24.0)	73 (35.1)
Basketball	12 (9.9)	26 (12.5)
Lacrosse	19 (15.7)	29 (13.9)
Soccer	44 (36.4)	59 (28.4)
Softball	7 (5.8)	9 (4.3)
Other	1 (0.8)	1 (0.5)
Fencing	9 (7.4)	11 (5.3)
Total	121	208

* Overall chi-square *p* = 0.39; *p* = 0.93 with one recording per participant; several cells had small counts.

## Data Availability

The data presented in this study are available on request from the corresponding author. The data are not publicly available due to privacy considerations, as the dataset contains breath samples collected from collegiate athletes whose demographic information (age, gender, and athletic program) could potentially enable re-identification within a limited university population.
